# Accuracy and Determinants of Radiographic Diagnosis in Pediatric Hand Fractures

**DOI:** 10.7759/cureus.90628

**Published:** 2025-08-20

**Authors:** Tala Thammaroj, Surut Jianmongkol, Nipaporn Tewattanarat, Anupol Panitchote, Punthip Thammaroj

**Affiliations:** 1 Department of Orthopedic Surgery, Faculty of Medicine, Khon Kaen University, Khon Kaen, THA; 2 Department of Radiology, Faculty of Medicine, Khon Kaen University, Khon Kaen, THA; 3 Division of Critical Care Medicine, Department of Medicine, Faculty of Medicine, Khon Kaen University, Khon Kaen, THA

**Keywords:** accuracy, child, fractures, hand, radiograph

## Abstract

Purpose: To investigate the accuracy of radiographic diagnosis of pediatric hand fractures and factors that contribute to diagnostic errors.

Methodology: We retrospectively reviewed cases of pediatric hand injuries in Srinagarind Hospital, Thailand, from January 2019 to December 2021. A total of 350 patients with accessible radiographs were interpreted by junior/senior radiology trainees, a pediatric/a senior radiologist, and two senior orthopedists. Age, sex, hand side, fracture type, and injury mechanism were collected.

Results*: *Among 167 patients with fractures, 55 (32.9%) had multiple fractures. For missed fractures (103 instances), junior radiology trainees missed more frequently than pediatric radiologists, with 35 (34%) vs. 8 (7.8%) instances (*P* = 0.004). Overall accuracy in the interpretation of different fracture types was 100% for greenstick, 77.4% for torus buckle, 76% for simple fractures. The pediatric radiologist showed the highest accuracy in the diagnosis of Salter-Harris type compared to others, 49 (28.7%) vs. 25-33 (14.6%-19.3%) (*P* = 0.001). On univariable analysis, vehicle injury mechanism was a borderline negative factor associated with missed fractures (odds ratio (OR), 0.38; 95% confidence interval (CI), 0.14-1.01; *P* = 0.05).

Conclusions: Our findings suggest that review by experienced pediatric radiologists is associated with higher diagnostic accuracy for pediatric hand fractures, particularly Salter-Harris injuries. Prospective studies are needed to determine whether incorporating senior review into routine workflows reduces missed diagnoses.

## Introduction

Fractures are common in pediatrics, and hand fractures represent one of the most frequent reasons for emergency department visits, accounting for one-fifth of all pediatric fractures [1,2]. While pediatric hand injuries usually result from minor trauma or sports-related incidents, hand fractures in adults are more often caused by high-energy mechanisms such as motor vehicle accidents. Radiographs of pediatric hand fractures differ from those of adults because of ongoing growth and development, incomplete ossification, susceptibility of growth plates to injuries, thicker periosteum, and high elasticity of children’s bones, which lead to incomplete rather than complete fractures. Accurate diagnosis depends on radiographic technique, the expertise of orthopedists and radiologists, the presence of overlying artifacts, and the specific fracture types in children. Because radiography remains the key investigation to screen, diagnose, and characterize pediatric fractures, its results directly impact patient care, further investigations, and dictate treatment decision-making [3-6]. Misdiagnosis will lead to unenviable complications or unnecessary healthcare costs for these pediatric fracture patients [4,7].

To reduce misdiagnosis of the pediatric hand fracture, good knowledge and awareness of the interpretation of pediatric hand fractures are essential [8-10]. Until now, few studies have systematically evaluated the diagnostic accuracy of radiographic diagnosis for hand fractures in children or examined the factors that influence diagnostic performance. This study aimed to (1) determine the diagnostic accuracy of radiographic interpretation of pediatric hand fractures among interpreter groups with varying levels of experience, and (2) identify patient, injury, and fracture characteristics associated with missed diagnoses.

## Materials and methods

**Figure 1:**
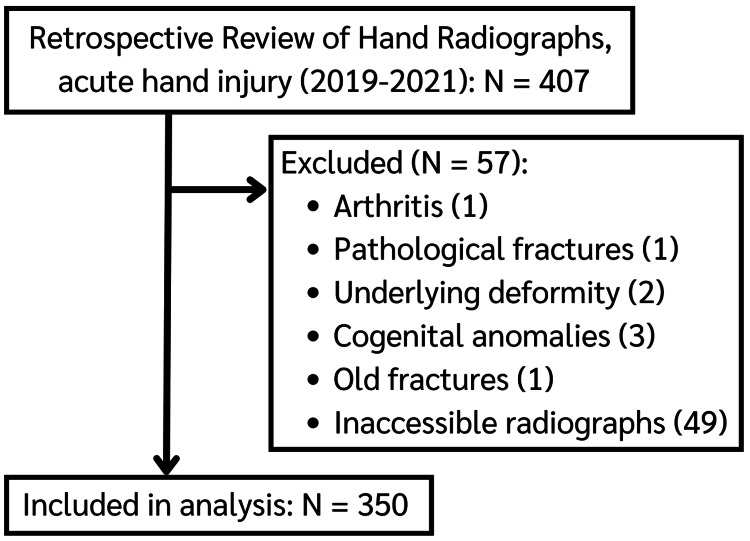
Flow diagram of the study population.

We conducted a retrospective review of 407 consecutive hand radiographs from January 2019 to December 2021 of pediatric patients aged 17 years or younger with acute pediatric hand injury and suspected fracture in an emergency room in Srinagarind Hospital, Khon Kaen University, Thailand. We excluded the patients with hand radiographs showing arthritis (1), pathological fractures (1), underlying hand deformity (2), congenital hand anomalies (3), old fractures (1), or inaccessible radiographs (49). Finally, a total of 350 cases were included, as shown in Figure 1. All hand radiographs (either hand, wrist, or finger) of at least two orthogonal views (posteroanterior, oblique, lateral projections) were evaluated. The radiographs were acquired using Samsung-iQuiaTM GC85A and Hitachi Radnext 80 digital radiography systems with exposure parameters (kVp and mAs) adjusted according to patient age and hand size to maintain diagnostic image quality while minimizing radiation exposure by the as low as reasonably achievable (ALARA) principle. Typical exposure settings in children ranged from 45-50 kVp and 2-3 mAs. Deviations from the standard protocol included referral outside hospital radiographs, modified positioning for uncooperative or immobilized patients, or acquisition of additional projections for suspected. 

Medical records were reviewed, and the data were recorded in a standard form. The data included age at the time of injury, sex, presence or absence of fracture, injured hand side, type of fracture, and the cause of injury (sport-related, falling, vehicles, abuse, direct impact).

Radiographic analysis

All radiographs were interpreted by five individual groups: (1) two 10-year experienced orthopedic (hand) surgeons, (2) one 10-year experienced radiologist, (3) one 7-year experienced pediatric radiologist, (4) 11 senior trainees of radiology, and (5) 21 junior trainees of radiology. In our institute, the radiology training program lasts three years and three months for the pediatric radiology rotation. We categorized trainees as junior if they had one or two years of experience and as senior if they had more than two to three years of experience. In groups with multiple evaluators, a total of 350 cases were divided and distributed proportionally in chronological order. The cases were read independently. The radiographs were reviewed via Picture Archiving and Communication System (PAC), with anonymized numbers in an unidentifiable way, and the interpreters were blinded to the final diagnosis and the radiologic reports in the first place, which were reported by trainees or radiologists.

Radiographs were evaluated in terms of the presence or absence of fracture, the side of the injured hand (right or left), the number of fractures (single or multiple), the site of fractures (distal radius, distal ulna, carpal bones, first to fifth metacarpals, proximal phalanges, middle phalanges, and distal phalanges), and the type of fractures (greenstick fracture, torus or buckle fracture, bow fracture, hairline fracture, simple fracture, segmental fracture, comminuted fracture, corner or bucket-handle fracture, and epiphyseal plate injury). The reference standard as a final diagnosis was given by (1) final post-treatment record for present or absent fracture, (2) a radiographic consensus by senior orthopedists together with senior radiologists who were blinded to the final post-treatment record. In case of a radiographic consensus for the type of fracture was deviated from a final post-treatment record, or not completely documented in the record. We chose a final diagnosis by consensus. Additionally, for present or absent fractures, all consensus by senior orthopedists together with senior radiologists was the same as all final post-treatment records. There were approximately three months elapsed between the initial radiograph interpretations and the consensus reviews among the senior orthopedists together with senior radiologists. When the radiographic diagnosis by investigators was the same as or different from the reference standard, it was considered a correct diagnosis or a misdiagnosis (either a missed fracture (false-negative) or an overdiagnosis (false-positive)), respectively.

Study data were collected and managed using Research Electronic Data Capture (REDCap). Written informed consent was obtained for participation in the study. The study was conducted by the Declaration of Helsinki and with approval by the Human Research Ethics Committee of Khon Kaen University (HE651181).

Statistical analysis

 The associations between staff positions and the proportion of misdiagnosed fractures, including missed and overdiagnosed fractures, were evaluated. For patients who had fractures, the proportion of missed fractures for each staff position was calculated by dividing the number of missed fractures by the total number of missed fractures across all staff positions. Similarly, for patients who did not have fractures, the proportion of overdiagnosed fractures for each staff position was determined by dividing the number of overdiagnosed fractures by the total number of overdiagnosed fractures across all staff positions. We used the Chi-square test and the Mann-Whitney U test to assess the statistical significance of the observed differences. For multiple comparisons between Interpreters for the number of missed or overdiagnosed fractures and types of fractures, we used pairwise comparisons between pairs of proportions with correction for multiple testing. To identify the factors that caused missed fractures, a univariable logistic regression analysis was conducted. Variable selection for the multivariable model used variables for the *P*-value < 0.1. To evaluate the agreement among multiple raters, Fleiss' Kappa coefficient was used. In our study, the analysis was conducted at the patient level, not the fracture level. For patients with multiple fractures, these were considered collectively as a single case for diagnostic accuracy assessment and analysis of influencing factors. All statistical analyses were carried out using the R software, version 4.3.1. We considered the result with a *P*-value < 0.05 (two-tailed) to be statistically significant.

## Results

A total of 350 pediatric patients with acute hand injuries suggestive of fracture were evaluated. Among these, 167 (47.7%) were confirmed to have fractures. The age group of 13-17 years had the highest proportion of hand injuries, accounting for 147 (42%) patients, followed by the age group of 6-12 years with 134 (38.3%) patients and 0-5 years with 69 (19.7%) patients. Males were 249 (71.1%), and females were 101 (28.9%). Left-hand injuries (191, 54.6%) were slightly more prevalent than those of the right-hand (160, 45.7%). The three predominant mechanisms of hand injuries were falls (158, 45.1%), followed by vehicles (111, 31.7%), and sport-related injuries (55, 15.7%), as detailed in Table 1.

**Table 1:** Demographic characteristics.

Demographic data	Number (%)
Age group (years)	
0-5	69 (19.7)
6-12	134 (38.3)
13-17	147 (42)
Sex	
Male	249 (71.1)
Female	101 (28.9)
Injured hand side	
Left	191 (54.6)
Right	160 (45.7)
Mechanism of injuries	
Falling	158 (45.1)
Vehicles	111 (31.7)
Sport-related	55 (15.7)
Direct impact	24 (6.9)
Abuse	2 (0.6)

Fracture characteristics

**Figure 2:**
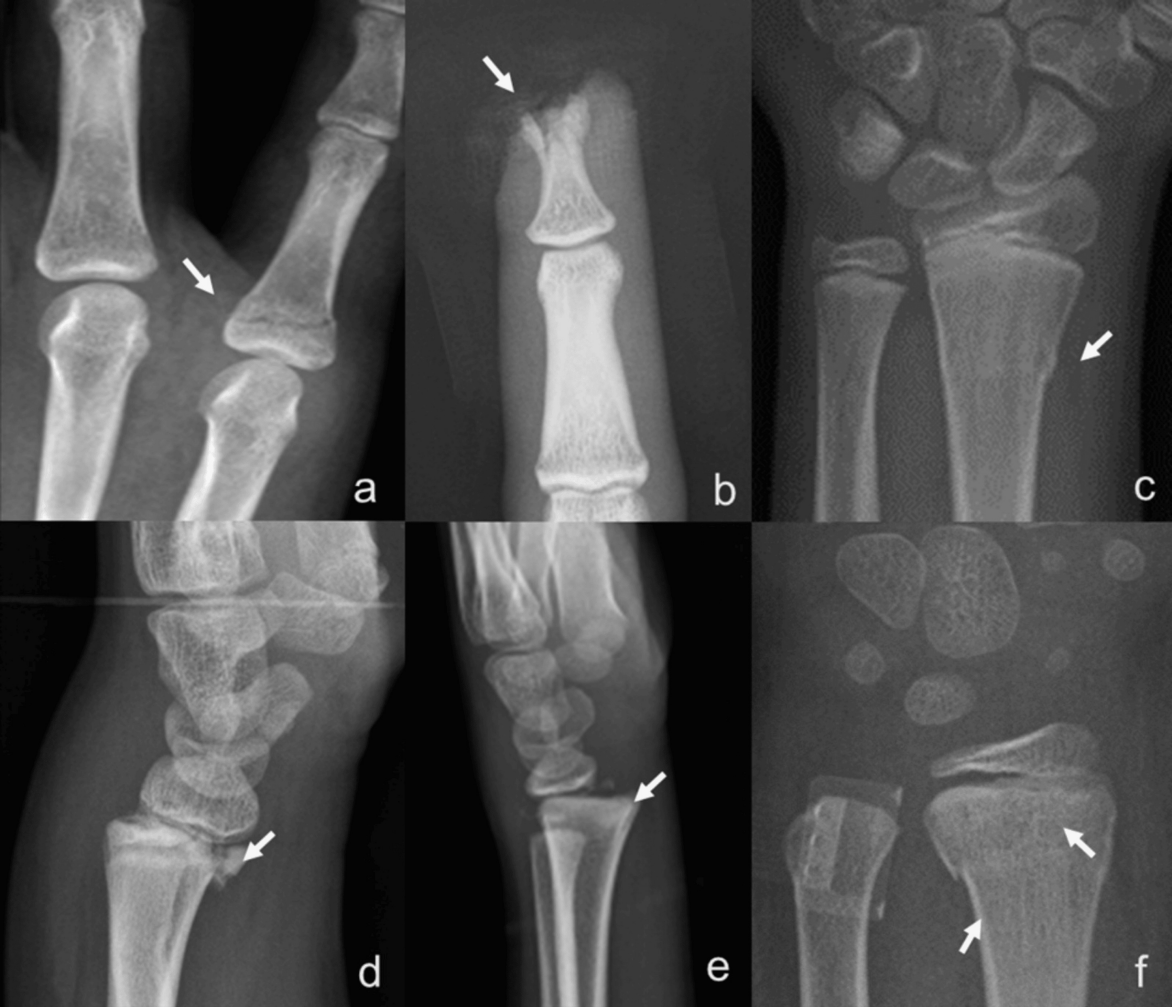
Types of fractures: (a) simple fracture, (b) comminuted fracture, (c) torus (buckle) fracture, and (d-f) Salter-Harris fractures.

In this study, among 167 patients with documented fractures, 55 (32.9%) patients presented with multiple fractures. The distal radius was the most frequently fractured site, observed in 108 (30.9%) patients, followed by the distal ulna in 45 (12.9%) patients, phalanges in 40 (11.4%) patients, and metacarpal bones in 17 (4.8%) patients. Fractures of the carpal bones were the least commonly reported. Among various types of fractures, the simple fracture was most prevalent, accounting for 67 (40.1%), followed by Salter-Harris fracture in 52 (31.1%) patients, torus buckle fracture in 50 (29.9%) patients, comminuted fracture in 16 (9.6%) patients, and greenstick fracture in 1 (0.6%) patient, as detailed in Table 2. Examples of types of fractures are shown in Figure 2. 

**Table 2:** Fracture characteristics.

Fracture characteristics	Number (%)
Site of fracture	
Distal radius	108 (30.9)
Distal ulna	45 (12.9)
Phalanges	40 (11.4)
Metacarpal bones	17 (4.86)
Carpal bones	4 (1.14)
Type of fractures	
Simple	67 (40.1)
Salter-Harris	52 (31.1)
Torus buckle	50 (29.9)
Comminute	16 (9.6)
Greenstick	1 (0.6)

Imaging interpretation

For the interpretation of misdiagnosis of fractures (missed and overdiagnosed fractures), the significant findings are as follows:

Missed fracture: Missed fracture occurred in 103 instances. Junior radiology trainees missed fractures more frequently, 35/103 (34%) instances, than did pediatric radiologists (8/103, 7.8%) (*P* = 0.004).

Overdiagnosed fracture: Overdiagnosed fracture was observed in 145 instances. Senior as well as junior radiology trainees reported overdiagnosed fractures more frequently than did senior orthopedists, senior radiologists, and pediatric radiologists. Specifically, senior and junior radiology trainees made overdiagnoses in 67 (46.2%) and 60 (41.4%) instances, respectively. In contrast, senior orthopedists, senior radiologists, and pediatric radiologists made overdiagnoses in 3 (2.1%), 7 (4.8%), and 8 (5.5%) instances, respectively (*P* < 0.001).

Missed multiple fractures: Multiple fractures were observed in 74 instances. Junior and senior radiology trainees made omissions frequently, with 30 (40.5%) and 20 (27.0%) instances, respectively, which were significantly higher than those of pediatric radiologists (5, 6.8%) or senior radiologists (7, 9.5%) (*P* < 0.001).

Overdiagnosed multiple fractures: Among 42 instances of overdiagnosed multiple fractures, senior and junior radiology trainees made 14 (33.3%) instances each, in contrast to 2 (4.8%) for the senior orthopedists (*P* = 0.01).

Overdiagnosed distal radius fracture: Overdiagnosis of distal radius fracture was made in 73 instances. Both senior and junior radiology trainees exhibited higher frequencies (*P *< 0.001) of overdiagnosis, 40 (54.8%) and 25 (34.2%), respectively, compared to pediatric radiologists (3, 6.2%), senior radiologists (2, 2.7%), and senior orthopedists (1, 1.4%).

Missed distal ulnar fracture: Among 66 instances of missed distal ulnar fracture cases, 18 (27.3%) and 27 (40.9%), respectively, were by the senior and junior radiology trainees, which were significantly (*P *< 0.001) higher than that of the pediatric radiologist (4, 6.1%).

Overdiagnosed distal ulnar fracture: For 48 instances of overdiagnosed distal ulnar fractures, senior and junior radiology trainees were responsible for 22 (45.8%) and 14 (29.2%) cases, respectively, whereas overdiagnosis of this type of fracture by senior orthopedists was only 2 (4.2%) (*P *< 0.001).

Overdiagnosed metacarpal fracture: For the 35 instances of overdiagnosed metacarpal fractures, senior and junior radiology trainees overdiagnosed this fracture with the counts of 13 (37.1%) and 18 (51.4%), respectively, whereas pediatric radiologist, senior radiologist, and senior orthopedists overdiagnosed this type of fracture only 0 (0%), 2 (5.7%), and 2 (5.7%), respectively (*P *< 0.001).

Missed proximal phalanx fracture: Among 34 cases of missed proximal phalanx fracture, senior and junior radiology trainees overlooked this type of fracture at 12 (35.3%) and 10 (29.4%) occasions, while pediatric radiologists made no mistakes (0, 0%) (*P *= 0.005).

Overdiagnosed proximal phalanx fracture: Of the 32 instances of overdiagnosis of proximal phalanx fractures, both senior and junior radiology trainees had higher frequencies, 12 (37.5%) and 15 (46.9%), respectively, compared with senior radiologists (1, 3.1%) (*P* < 0.001).

All these findings and more details are given in Table 3.

**Table 3:** Interpreters and numbers of missed or overdiagnosed fractures. ^*^P < 0.05 compared with pediatric radiologists. ^**^P < 0.05 compared with senior radiologists. ^***^P < 0.05 compared with senior orthopedists. Dx, diagnosis; Fx, fracture

Interpretation	Senior orthopedists, *n* (%)	Senior radiologist, *n* (%)	Pediatric radiologist, *n* (%)	Senior radiology trainees, *n* (%)	Junior radiology trainees, *n* (%)	*P*-value
Missed Fx (*n* = 103)	22 (21.4)	16 (15.5)	8 (7.8)	22 (21.4)	35 (34)^*^	0.003
Over Dx Fx (*n* = 145)	3 (2.1)	7 (4.8)	8 (5.5)	67 (46.2)^*, **, ***^	60 (41.4)^*, **, ***^	<0.001
Missed multiple Fx (*n* = 74)	12 (16.2)	7 (9.5)	5 (6.8)	20 (27.0)^*^	30 (40.5)^*^,^**^	<0.001
Over Dx multiple Fx (*n* = 42)	2 (4.8)	5 (11.9)	7 (16.7)	14 (33.3)^***^	14 (33.3)^***^	0.005
Missed distal radius Fx (*n* = 61)	12 (19.7)	11 (18)	6 (9.8)	11 (18)	21 (34.4)	0.096
Over Dx distal radius Fx (*n* = 73)	1 (1.4)	2 (2.7)	5 (6.8)	40 (54.8)^*, **, ***^	25 (34.2)^*, **, ***^	<0.001
Missed distal ulna Fx (*n* = 66)	12 (18.2)	5 (7.6)	4 (6.1)	18 (27.3)^*^	27 (40.9)^*^,^**^	<0.001
Over Dx distal ulna Fx (*n* = 48)	2 (4.2)	7 (14.6)	3 (6.2)	22 (45.8)^*, ***^	14 (29.2)^***^	<0.001
Missed carpal bone Fx (*n* = 9)	3 (33.3)	0 (0)	1 (11.1)	3 (33.3)	2 (22.2)	0.173
Over Dx carpal bone Fx (*n* = 12)	0 (0)	1 (8.3)	3 (25.0)	4 (33.3)	4 (33.3)	0.250
Missed metacarpal bone Fx (*n* = 14)	4 (28.6)	3 (21.4)	0 (0)	5 (35.7)	2 (14.3)	0.153
Over Dx metacarpal bone Fx (*n* = 35)	2 (5.7)	2 (5.7)	0 (0)	13 (37.1)^*, **, ***^	18 (51.4)^*, **, ***^	<0.001
Missed Dx prox phalanx Fx (*n* = 34)	6 (17.6)	6 (17.6)	0 (0)	12 (35.3)^*^	10 (29.4)^*^	0.005
Over Dx prox phalanx Fx (*n* = 32)	2 (6.2)	1 (3.1)	2 (6.2)	12 (37.5)^**^	15 (46.9)^*, **, ***^	<0.001
Missed Dx middle phalanx Fx (*n* = 15)	1 (6.7)	2 (13.3)	3 (20.0)	3 (20.0)	6 (40.0)	0.305
Over Dx middle phalanx Fx (*n* = 13)	0 (0)	0 (0)	0 (0)	7 (53.8)	6 (46.2)	0.000
Missed Dx distal phalanx Fx (*n* = 14)	3 (21.4)	0 (0)	1 (7.1)	4 (28.6)	6 (42.9)	0.033
Over Dx distal phalanx Fx (*n* = 20)	1 (5)	4 (20)	4 (20)	7 (35)	4 (20)	0.381

Accuracy of fracture type interpretation

The overall accuracy in the interpretation of different fracture types by all interpreters was as follows: 100% for greenstick fracture, 77.4% for torus buckle fracture, 76% for simple fracture, 75% for bow fracture, 69.8% for Salter-Harris fracture, and 62.8% for comminuted fractures. For the accuracy of the interpretation of each type of fracture, the significant findings are as follows;

Torus Buckle fracture: Senior and junior radiology trainees showed a lower accuracy rate compared to pediatric radiologists, with respective accuracies of 39 (17.3%) and 35 (15.5%) vs. 58 (25.7%) (*P *= 0.001).

Simple fracture: Pediatric radiologists and junior radiology trainees showed lower accuracy rates in interpretation, 52 (19.3%) and 46 (17.1%), respectively, compared with senior radiologists, 66 (24.5%) (*P* = 0.002).

Salter-Harris fracture: This type of fracture appeared to be particularly difficult to diagnose accurately; among all examiners, pediatric radiologists achieved better results, 49 (28.7%), compared with senior and junior radiology trainees, senior radiologists, and senior orthopedists, whose accuracies were 31 (18.1%), 33 (19.3%), 33 (19.3%), and 25 (14.6%), respectively (*P* = 0.001).

More details of these findings are summarized in Table 4.

**Table 4:** Interpreters and accuracy of fracture type interpretation. ^*^P < 0.05 compared with pediatric radiologists. ^**^P < 0.05 compared with senior radiologists. ^***^P < 0.05 compared with senior orthopedists. Accuracy = proportion of the number of accurate diagnoses per total correct interpretation for each fracture type. CI, confidence interval

Correct interpretation of the type of fracture	All interpreters % (95% CI)	Senior orthopedists, *n* (%)	Senior radiologist, *n* (%)	Pediatric radiologist, *n* (%)	Senior radiology trainees, *n* (%)	Junior radiology trainees, *n* (%)	*P*-value
Simple (*n* = 269)	76% (71.1-80.3)	55 (20.4)	66 (24.5)	52 (19.3)^**^	50 (18.6)	46 (17.1)^**^	0.002
Torus buckle (*n* = 226)	77.4% (72.1-82)	45 (19.9)	49 (21.7)	58 (25.7)	39 (17.3)^*^	35 (15.5)^*^	0.001
Salter-Harris (*n* = 169)	69.5% (63.3-75.2)	25 (14.8)^*^	31 (18.3)^*^	49 (29)	31 (18.3)^*^	33 (19.5)^*^	0.001
Comminute (*n* = 49)	62.8% (51.1-73.3)	9 (18.4)	9 (18.4)	15 (30.6)	9 (18.4)	7 (14.3)	0.128
Greenstick (*n* = 5)	100% (46.3-100)	1 (20)	1 (20)	1 (20)	1 (20)	1 (20)	0.990

The inter-rater agreement, as measured by the Kappa coefficient, for the interpretation of fractures among senior orthopedists, senior radiologists, and pediatric radiologists was 0.801 (95% confidence interval (CI), 0.75-0.85, *P *< 0.001). This indicates substantial agreement among these professionals. The inter-rater agreement for the interpretation of fractures among the senior and junior radiology trainees was 0.465 (95% CI 0.35-0.55, *P *< 0.001). This indicates moderate agreement among these trainees.

Factors associated with missed fractures and overdiagnosed fractures based on demographic characteristics

In the univariable analysis examining factors associated with missed fractures based on demographic characteristics, age was not significantly associated with missed fractures (odds ratio (OR) 0.97; 95% CI 0.89-1.06, *P *= 0.531). Similarly, gender showed no significant relationship (OR 1.44; 95% CI 0.66-3.36, *P *= 0.37). Regarding the mechanism of injury, falling (OR 1.06; 95% CI 0.43-2.67, *P* = 0.90) and vehicle-related injuries (OR 0.38; 95% CI = 0.14-1.01, *P *= 0.05) displayed no definitive association, while direct impact had an OR of 0 with an immeasurable CI. Furthermore, age was significantly associated with overdiagnosed fractures (OR 1.08; 95% CI 1.02-1.15, *P *= 0.008). In contrast, gender and mechanisms of injury were not associated with overdiagnosed fractures, as detailed in Table 5.

**Table 5:** Univariable logistic regression analysis of factors associated with missed fractures and overdiagnosed fractures based on demographic characteristics. OR, odds ratio; CI, confidence interval; NA, not applicable

Factors	Missed fractures		Overdiagnosed fractures	
	OR (95% CI)	*P*-value	OR (95% CI)	*P*-value
Age	0.97 (0.89-1.06)	0.531	1.08 (1.02-1.15)	0.008
Boy	1.44 (0.66-3.36)	0.373	1.24 (0.66-2.3)	0.505
Causes				
Sport	1		1	
Falling	1.06 (0.43-2.67)	0.904	0.48 (0.19-1.15)	0.108
Vehicle	0.38 (0.14-1.01)	0.050	1.11 (0.39-3.05)	0.845
Direct impact	3.63 x 10^-8^ (NA-1.13 x 10^38^)	0.989	0.70 (0.17-2.94)	0.617
Abuse	-	-	0.5 (0.02-13.63)	0.638
Other	-	-	0.25 (0.01-2.95)	0.283

## Discussion

In our study, the prevalence of hand injuries was highest in the 13-17 year age group, accounting for 147 (42%) patients. About one-third of the patients with fractures had more than one fracture. The distal radius was the most frequently fractured site in 108 (30.9%) patients.

The interpretation of the pediatric hand fractures in our study revealed that the overdiagnosed fractures as well as the missed fractures were observed not only among the trainees, but also among the senior radiologists, pediatric radiologists, and senior orthopedists.

Missed fractures were commonly found in junior radiology trainees, but at a far lower frequency in pediatric radiologists. The overdiagnosis of fractures was more frequently detected in both senior and junior radiology trainees than in senior orthopedists, senior radiologists, and pediatric radiologists. As was expected, the missed and overdiagnosed fractures were more frequently made by the less-experienced interpreters. In the previous study of Chew and Chong [10], the actual incidence of hand fractures among children under 17 years of age with suspected hand fractures was very high (193/204, 94.6%), but, among 193 confirmed fracture cases, 16 cases (8.3%) were not diagnosed at the first radiographic examination. Similarly, Fitschen-Oestern et al. (2020), using data from the TraumaRegister DGU®, reported 727 cases (6.6%) of missed hand injuries in pediatric and adult patients. The most commonly missed injuries were 104 carpal fractures or dislocations (11.2%) [7]. To reduce diagnostic errors, we suggest that trainees interpret pediatric hand radiographs under the supervision of more experienced clinicians, particularly within the context of radiology education.

In this study, the common sites of missed fractures were the distal ulna (66), the distal radius (61), and the proximal phalanx (34) in order. On the other hand, the common sites of overdiagnosed fractures were the distal radius (73), the distal ulna (48), and the metacarpal (35) in order. Thus, we should pay special attention when we have patients with suspected fractures on these sites. From previous studies, there are various diagnostic clues to improve diagnostic accuracy, for example, clinical localization cues [11], interpretation of normal epiphyses by obtaining radiographs of the uninjured contralateral side for comparison, and true posteroanterior and lateral X-rays of the isolated digital fracture [10]. As was mentioned previously, these may help to identify the exact location in cases of suspected fracture, particularly when the fractures are not found on the standard plain radiographs only.

In the aspect of interpretation for a specific type of fractures, trainees tend to incorrectly interpret torus buckle and simple fractures compared to the experienced staff. Awareness to report these specific types of fractures is essential.

Salter-Harris-type fracture is difficult to diagnose and contributes 31.1% of all fractures in our study, which is comparable to the proportion reported in other studies (34%) [12]. As is expected, for a Salter-Harris type fracture, the pediatric radiologist gave the most accurate interpretation compared with the radiology trainees or other staff. Thus, deep knowledge and familiarity with the radiological anatomy of children’s hands are essential for accurate interpretation of this fracture type. Similar to the claims of other studies, to improve diagnostic accuracy, doctors should familiarize themselves with the location of epiphyses [10]. As the association between accurate reading of Salter-Harris fracture and expertise of pediatric radiologist was found, but a lack of adjustment for confounding without multivariable analysis, differences in accuracy could be partly explained by other factors, rather than interpreter experience alone. The recommendation for changing clinical workflow, such as more senior review, is plausible but should be framed as a hypothesis for further testing.

Regarding the factors affecting missed and overdiagnosed fractures, we analyzed the possible importance of age groups of patients, male gender, and mechanisms of injury, including sport injury, fall, vehicle, and direct impact injuries. Although they had no statistically significant association with missed fractures, the vehicle injury almost reached a significant negative factor associated with missed fractures on univariable analysis. This might be explained by the fact that this type of injury is a high-energy trauma, which may cause severe soft tissue and bone damage together, which can help the interpreter easier to detect or trace the fracture on plain radiographs. Or this vehicle injury association could be due to chance or confounding. For overdiagnosed fractures, there appears to be an association between increasing age in children and an overestimation of fracture presence. This finding also could be due to chance or confounding. Future research is needed. 

In our study, good agreements were observed among senior orthopedists, senior radiologists, and pediatric radiologists in their interpretations of all different sites of fractures. There was a moderate degree of agreement among trainees, which implied that there were several cases where a senior trainee's interpretation differed from a junior trainee's.

We performed power calculations using our results. The statistical power for missed fractures, overdiagnosed fractures, missed multiple fractures, and overdiagnosed multiple fractures was 95%, 100%, 99.6%, and 86.5%, respectively. However, the power was less than 50% for detecting missed and overdiagnosed fractures in specific bones like the carpal bone and metacarpal bones, as well as in the middle phalanx and distal phalanx. Additionally, the power was low for certain types of fractures, including simple, comminuted, and greenstick fractures.

The strengths of the study include (1) the study addresses a clinical gap with high relevance to orthopedics, radiology, and emergency medicine, particularly important given the unique anatomical complexities of pediatric imaging and the need for evidence-based strategies to minimize missed injuries, and (2) the relatively large dataset enhances the precision and accuracy estimates. Additionally, the multiple interpreter groups with defined experience levels allow exploration of expertise effects, (3) blinding interpreters were blinded to the final diagnosis and original radiology reports, reducing review bias, and d) standardized fracture classification using recognized pediatric fracture categories (greenstick, torus, Salter-Harris) enhances comparability with other literature. This study has some limitations: (1) the retrospective design (Thai tertiary-hospital single-center) with the results may not apply universally, (2) risk of incorporation bias in the reference standard, (3) lack of multivariable modeling that can get rid of confounding factors, and (4) radiographic interpretations were performed on an electronic platform, which may not accurately reflect the challenges of interpretation in real-time clinical settings.

## Conclusions

Our findings suggest that review by experienced pediatric radiologists is associated with higher diagnostic accuracy for pediatric hand fractures, particularly for Salter-Harris injuries. Prospective studies are needed to determine whether incorporating senior review into routine workflow reduces missed diagnoses.
